# Monitoring the responsiveness of T and antigen presenting cell compartments in breast cancer patients is useful to predict clinical tumor response to neoadjuvant chemotherapy

**DOI:** 10.1186/s12885-017-3982-1

**Published:** 2018-01-15

**Authors:** David A. Bernal-Estévez, Oscar García, Ramiro Sánchez, Carlos A. Parra-López

**Affiliations:** 1 Department of Microbiology, Graduated School in Biomedical Sciences, Universidad Nacional de Colombia, Bogotá, Colombia; 2 Servicio de seno y tejidos blandos, Instituto Nacional de Cancerología, Bogotá, Colombia; 3 Clínica del Seno, Bogotá, Colombia; 4 Immunology and Clinical Oncology Research Group (GIIOC), Fundación Salud de los Andes, Bogotá, Colombia

**Keywords:** Breast cancer, Chemotherapy, Neoadjuvant, T cells, Dendritic cells, Doxorubicin, Immune-monitoring

## Abstract

**Background:**

Vaccination of mice with tumors treated with Doxorubicin promotes a T cell immunity that relies on dendritic cell (DC) activation and is responsible for tumor control in vaccinated animals. Despite Doxorubicin in combination with Cyclophosphamide (A/C) is widely used to treat breast cancer patients, the stimulating effect of A/C on T and APC compartments and its correlation with patient’s clinical response remains to be proved.

**Methods:**

In this prospective study, we designed an in vitro system to monitor various immunological readouts in PBMCs obtained from a total of 17 breast cancer patients before, and after neoadjuvant anti-tumor therapy with A/C.

**Results:**

The results show that before treatment, T cells and DCs, exhibit a marked unresponsiveness to in vitro stimulus: whereas T cells exhibit poor TCR internalization and limited expression of CD154 in response to anti-CD3/CD28/CD2 stimulation, DCs secrete low levels of IL-12p70 and limited CD83 expression in response to pro-inflammatory cytokines. Notably, after treatment the responsiveness of T and APC compartments was recovered, and furthermore, this recovery correlated with patients’ residual cancer burden stage.

**Conclusions:**

Our results let us to argue that the model used here to monitor the T and APC compartments is suitable to survey the recovery of immune surveillance and to predict tumor response during A/C chemotherapy.

**Electronic supplementary material:**

The online version of this article (10.1186/s12885-017-3982-1) contains supplementary material, which is available to authorized users.

## Background

Pre-clinical experimental evidence suggests that tumor treatment with some chemo-radiotherapy regimens induce in tumor cells immunogenic cell death (ICD) that promotes the antigenicity and immunogenicity of tumors [1]. The immunogenicity of tumor cells dying via ICD is favored by cross-presentation of antigens by DCs to anti-tumor CD8 T-cells responsible for controlling the tumor. Retrospective studies have confirmed that cancer patients treated with Doxorubicin having mutations in molecular components involved in recognition of tumor cells that die by ICD have shorter overall survival and a higher risk of metastatic disease [2].

Clinical evidence on the immunogenicity of tumors induced by anti-tumor therapy has shown that a good clinical response to Doxorubicin is correlated with changes in immune contexture of the tumor [3, 4]. Furthermore, the study of biomarkers in colon cancer to predict clinical response has identified immunological signatures in the tumor microenvironment with predictive and prognostic value [4, 5]. The efforts to demonstrate a relationship between immunogenicity of tumors induced by chemotherapy and anti-tumor immune signatures in breast cancer (BC) patients with clinical response to treatment have yielded some evidence in this direction [6, 7]. Despite, these studies for the identification of biomarkers with the potential to predict chemotherapeutic responses in BC are encouraging, blood-based monitoring systems to predict clinical response to treatment does not exist. In the case of BC patients under neoadjuvant therapy, the identification of predictive markers of clinical response using whole blood or PBMCs is desirable because this would help the adjustment of the chemotherapy regimes in trying to achieve pathological complete responses (pCR) in all patients treated.

Tumor growth is the result of tumor escape of immune surveillance due to a poor performance of T and antigen presenting cell (APC) compartments [8]. Although experimental evidence suggests that primary chemotherapy with Doxorubicin induces ICD that favors anti-tumor responses and changes in the contexture of the tumor, the effect of Doxorubicin on T and APC compartments in patients under primary chemotherapy is yet to be demonstrated. We hypothesized that a favorable clinical response of BC tumors to neoadjuvant therapy with Doxorubicin and Cyclophosphamide (A/C) will revert suppression in these two compartments. In a recent study, we design an in vitro system to monitor the specific anti-tumor response before and after anti-tumor therapy [9]. Our results suggest that the status of disease-free survival and a complete clinical response is supported by tumor-specific T lymphocytes induced by anti-tumor treatment. To generate clinical evidence that chemotherapeutic agents inducing ICD restores immunosurveillance of the T and APC compartments in cancer patients with clinical tumor response to Doxorubicin, in the present work we studied a group of 17 patients with BC in neoadjuvant therapy (three cycles of A/C), whose tumors experienced significant clinical response after chemotherapy. This behavior of the tumor prompted us to investigate whether a favorable clinical response to primary chemotherapy (A/C) is correlated with the better performance of T cells and APCs interaction. To do this, we compared the immunological performance of T and APC compartments in peripheral blood of these patients before and after chemotherapy. We found that the overall suppression of these two compartments perceived before treatment is reversed after chemotherapy and this recovery correlates with clinical response. Altogether our results let us argue four things: first, the unresponsiveness to stimuli of T/APC compartments observed in these BC patients before treatment starts to recover after three cycles of A/C; second, primary chemotherapy reestablished the crosstalk between T/APC compartments; third, the recovery of this crosstalk is correlated with the clinical response of the tumor and, fourth, monitoring T/APC compartments may be useful to identify predictive biomarkers of tumor responsiveness to treatment.

## Methods

### Patients and blood samples

This prospective study was approved by the ethics committee of the Instituto Nacional de Cancerología - Bogotá (reference number ACT-018 May 2012). The patients and all healthy donors had signed an informed consent form before blood samples were taken. A total of 560 patients with pathological diagnosis of breast cancer were interviewed at the Instituto Nacional de Cancerología and the Clínica del Seno (Bogotá-Colombia) between 2012 to 2015; of these patients, 36% were eligible to be treated with Doxorubicin and Cyclophosphamide (A/C) scheme as neoadjuvant chemotherapy and 22% of total patients overexpress Her2/neu; a total of 17 patients with ductal invasive carcinoma (DIC) were included in the study. After informed consent had been signed, two blood samples were taken (20 mL each) one to three days before the first dose of chemotherapy and eight to ten days after third dose of A/C chemotherapy. Healthy women (HD), were used as controls (age-matched). PBMCs were isolated by density gradient with Ficoll Hypaque (GE) and cryopreserved in liquid nitrogen in freezing media (RPMI-1640 50%, FBS 40% and 10% of DMSO) until used. Clinical data of the included patients is shown in Table 1; clinical response was evaluated by residual cancer burden (RCB) clasification [10]. RCB was calculated based on primary tumor bed area, overall cancer cellularity, percentage of cancer that is in situ disease, number of positive lymph nodes and diameter of largest metastasis.

**Table 1:** Clinicopathologic factors of BC patients

Patients’ clinical characteristics	n
Age (years) mean 55.6	
<50	6
>50	11
TNM (stage)	
1	1
2	10
3	6
Clinical stage	
IIA	2
IIB	8
IIIA	6
IIIB	1
Clinical Lymph Node Classification	
cN0	3
cN1	10
cN2	4
cN3	0
Systemic metastases	
No	17
Yes	0
Residual Cancer Burden (RCB)*	
RCB-III	0
RCB-II	11
RCB-I	2
pCR	4
Estrogen receptor (ER)	
Positive (>10%)	13
Negative (<10%)	4
Progesterone receptor (PR)	
Positive (>10%)	10
Negative (<10%)	7
Ki-67	
Positive (>14%)	4
Negative (<14%)	13
Her2/neu	
Positive	5
Negative	12
Scarff-Bloom-Richardson (SBR) grade	
I	0
II	10
III	7
Breast Cancer sub-types**	
Luminal A	3
Luminal B	10
Triple negative	3
Her2/neu overexpressing	1

*Classification based on MD Anderson Center RCB calculator

**Luminal A: ER+ and/or PR+, HER-2− and Ki67 low. Luminal B: ER+ and/or PR+, HER-2− and/or HER-2+ and Ki67 high. Triple negative: ER− and PR− and HER-2−. Her2/neu overexpressing: ER- and Her2/neu+

### Flow cytometry

**Fig. 1:**
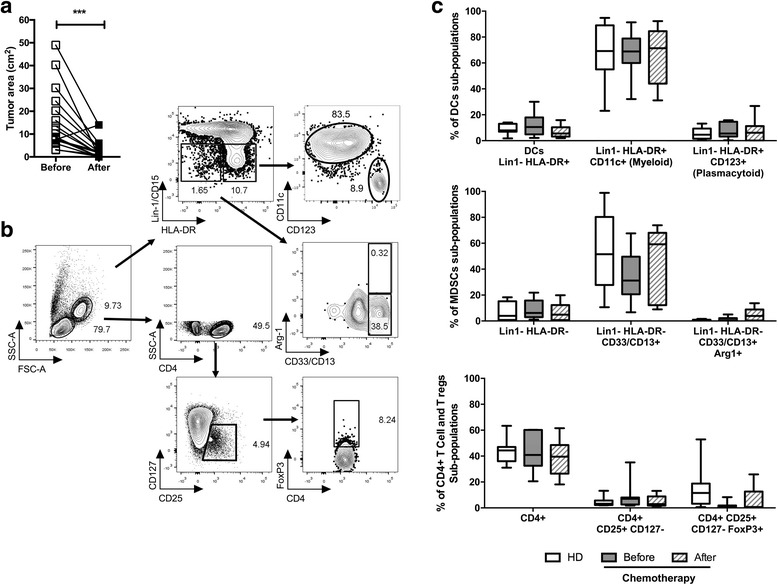
Assessing different cell populations ex vivo in PBMCs from healthy donors and BC patients before and after chemotherapy. **a** Paired analysis of tumor size (area in cm^2^) of the patients before therapy and after three cycles of A/C chemotherapy (*n* = 17). **b** Working strategy for multi-parametric cell analysis using flow cytometry. Monocytes and lymphocytes were defined by contour plots using SSC-A vs. FSC-A. Myeloid and plasmacytoid dendritic cell (DCs) (cells HLA-DR+ Lin1/CD15- CD11c + or HLA-DR+ Lin1/CD15- CD123+ respectively) and myeloid-derived suppressor cells (MDSCs) (cells HLA-DR- Lin1/CD15- CD13+ CD33+ ± Arginase 1+), were analyzed within the monocytic cell region. Finally, the percentage of CD4+ and regulatory T cells CD4+ CD25+ CD127- FoxP3+ (Tregs) was estimated within the lymphoid cell region. **c** Percentage of different sub-populations ex vivo in PBMCs from healthy donors (white box *n* = 10) and BC patients before (gray box *n* = 12) and after chemotherapy (dashed box *n* = 12). Panels summarize the percentages of DCs populations (top panel), MDSCs (middle panel) and CD4+ and CD4+ Tregs: CD4+ CD25+ CD127- and CD4+ CD25+ CD127- FoxP3+ (middle and right panels at the bottom). Paired analysis by Wilcoxon test, *** *p* < 0.001. Box and whiskers graph with 10–90% of data

**Fig. 2:**
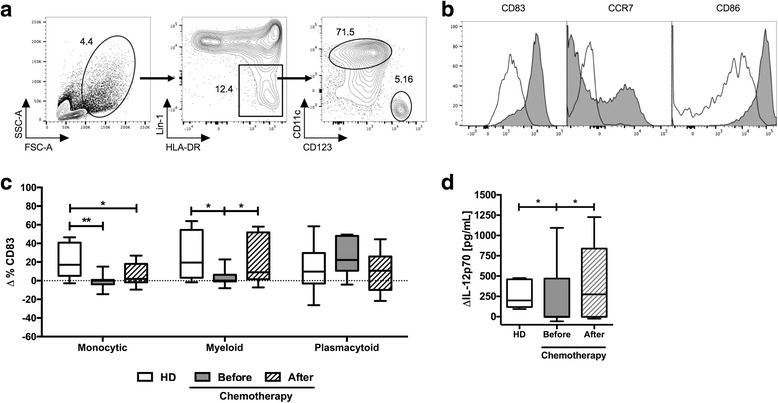
DC maturation and IL-12p70 production are hampered in cancer patients before treatment. **a** The analysis by contour plots of a representative sample of myeloid (HLA-DR+ Lin1- CD11c+) and plasmacytoid (HLA-DR+ Lin1- CD123+) DCs is shown. **b** Representative histograms comparing the phenotype (CD83, CCR7, and CD86) of immature (empty histogram) and mature (gray histogram) in myeloid DCs (HLA-DR+ Lin1- CD11c+) derived from HD. **c** Quantification of CD83 expression in response to maturation stimulus (delta of the percentage of CD83 expression between mature and immature DCs) in DCs derived in PBMCs from HD (white box), and breast cancer patients before and after chemotherapy (grey and dashed box, respectively) in monocytic cells (defined by FSC-A vs SSC-A (left)), myeloid (HLA-DR+ Lin1- CD11c + (middle)) or plasmacytoid DCs (HLA-DR+ Lin1- CD123+ (right)). **d** Delta of concentration in pg/mL of IL-12p70 secreted in culture supernatants (difference in concentration secreted by mature and immature) DCs from HD (white box, *n* = 10) and patients before (gray box, *n* = 17) and after chemotherapy (dashed box, *n* = 17). Boxes and whiskers 10–90%, two-way ANOVA analysis, with Turkey’s multiple comparison tests, * *p* < 0.05, ** *p* < 0.01

For the analyses of different sub-populations and phenotype of T and APC we use specific staining panels. For ex vivo sub-populations in the PBMCs obtained from patients before and after treatment and HD we quantified in a single tube: (i) regulatory T cells, (ii) Myeloid-derived suppressor cells (MDSCs), and (iii) myeloid DCs and plasmacytoid DCs by the combination of the following antibodies: CD4-BV510, CD25-APC-Cy7, CD127-PECy5, FoxP3-Pacific Blue, Lin1-FITC (CD3, CD14, CD16, CD19, CD20, CD56), CD15-FITC, CD13-PE, CD33-PE, HLA-DR-PE Dazzle 594, CD11c-Alexa Fluor 700, CD123-PECy7 (all from Biolegend); and Arg1-APC (R&D Systems). The gating strategy for ex vivo subpopulations is depicted in Fig. 1b. For the phenotype of mature DCs, the following antibodies were used: Lin1-FITC, HLA-DR-PE Dazzle 594, CD11c-Alexa Fluor 700, CD123-PECy7, CD83-PECy5, CCR7-Alexa Fluor 647, and CD86-PE (all from Biolegend), the gating strategy is depicted in Fig. 2a. For the analysis for TCR internalization and T cell activation markers, the following antibodies were used: CD3-FITC, CD154-APC, CD69-PECy7, CD25-PE (all from Biolegend). Finally, for cytokine secretion, we measure in the supernatant by Cytometric Bead Array (CBA) human Th1/2 and inflammatory cytokines (BD) of DCs after maturation and T cell activation. Flow cytometry data was acquired using FACS Aria II (BD) and analyzed using FlowJo Software (Tree Star Inc.).

### Functionality of APC and T cell compartments

**Fig. 3:**
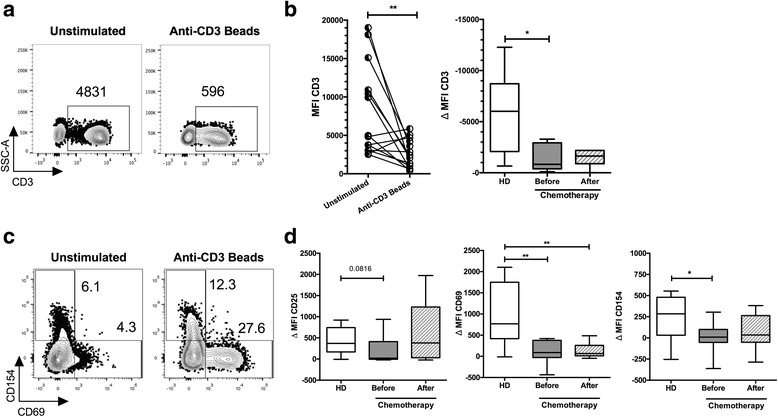
Suppressed T cell responsiveness in BC patients before chemotherapy. **a** Representative contour plots of T cells (SSC-A vs. CD3+) of HD unstimulated or stimulated 24 h with anti-CD3/CD28/CD2 beads (numbers represent MFI of CD3). **b** Quantification of CD3 MFI in PBMCs of HD in response to in vitro stimulation (left panel), and delta of CD3 MFI from HD (white box), and BC patients before (gray box) and after chemotherapy (dashed box – right panel). **c** Representative contour plots of T cell (gated on CD3+) activation phenotype (CD154 vs. CD69) of cells obtained from HD in response to in vitro stimulation with anti-CD3/CD28/CD2 beads, numbers represent the percentage of each population. **d** Quantification of MFI of each activation marker (delta of stimulated minus unstimulated cells) of CD25 (left panel), CD69 (middle panel) and CD154 (right panel) of HD (white box), and BC patients before (gray box) and after chemotherapy (dashed box). Box and whiskers 10–90%. HD (*n* = 12), patients before (*n* = 17) and after chemotherapy (*n* = 17). Non-parametric *t*-test (panel B – left panel) and Two-way ANOVA analysis, with Turkey’s multiple comparison tests, * p < 0.05, ** p < 0.01

The phenotype and functional capacity of monocyte-derived DCs was evaluated in vitro after exposure of PBMCs to IL-4 and GM-CSF as described by Martinuzzi et al. [11], and maturated with pro-inflammatory cytokines in combination with Type I interferons as described by Mailliard et al. [12, 13]. Briefly, after induction of immature DCs (iDCs), a combination of IFN-γ (R&D systems), IFN-α (Intron-A- ROCHE), TNF-α, IL-6, IL-1β (all from Cellgenix), and Poly I:C (Sigma-Aldrich). For DCs maturation phenotype, the expression of CD11c+, HLA-DR+ and Lin1- was analyzed by flow cytometry (FC) in total PBMCs (Fig. 2a), and secretion of IL-12p70 was quantified by CBA (BD Biosciences) in the supernatant of mature DCs culture. Simultaneously, for the determination of responsiveness of T cells, 5 × 10^6^ PBMCs/mL were stimulated 24 h with a mixture of anti-CD3, CD28, and CD2 microbeads (Miltenyi Biotec) in a ratio 2:1 (PBMC:beads) cultured in AIM-V media (Thermo Fisher Scientific). After stimulation, we quantify the internalization of TCR (reduction of CD3 MFI) and expression of CD69, CD25 and CD154 (MFI and percentage) in CD3+ T cells as shown in Fig. 3a and c.

### Statistical analysis

All immunological data were normalized against unstimulated controls (delta between stimulated T cells or mature DCs with unstimulated T cells or iDCs respectively). Since most of the readouts did not present a normal distribution, non-parametric tests were applied to test for statistical differences between groups with Two-way ANOVA with Turkey’s multiple comparison tests. For paired samples, we used Wilcoxon test. Receiver Operating Characteristic (ROC) curves analysis were done with Prism 5 software (GraphPad). Factorial Analysis with Principal Component Analysis (PCA) was made in SPSS (IBM – version 20), the component matrix was rotated using Varimax rotation to facilitate the interpretation of PCA, two principal components were extracted, and PC loadings of variables and PC scores of samples were a plot in a two-dimensional graph. The multifactorial categorical analysis was done using Generalized estimating equations (GEE) in STATA 13 software [14].

## Results

### After three cycles of antitumor therapy, the levels of different populations of suppressor cells do not undergo significant changes in the peripheral blood

Neoadjuvant anti-tumor therapy with A/C is used in BC patients to induce a reduction in the tumor size before surgery. It is well known that anti-tumor therapy with A/C can produce a clinical response evidenced by the reduction in RCB in most patients. The monitoring of tumor response in 17 patients with BC during neoadjuvant chemotherapy showed that after treatment pathological complete response (pCR) observed in four of 17 patients (based on RCB index), with a tumor shrinkage in 16/17 patients who experienced a significant reduction in tumor area (Fig. 1a). Based on tumor response in this cohort, we explored the behavior of different immunological readouts before and after chemotherapy that could be associated with the clinical response. It is well known that tumor escape from immunosurveillance is favored by infiltration of the tumor by different populations of suppressor cells such as CD4+ CD25+ FoxP3+ regulatory (Tregs) [15], suppressor macrophages [16], Myeloid Derived Suppressor Cells (MDSCs) [17], and immature DCs, that inhibit tumor-specific T cells favoring escape from immune surveillance. Different reports indicate that the expansion in blood of some of these cells is a common finding in patients with BC [18–20], and only certain reports explore how anti-tumor therapy modulates the blood levels of these cells in colorectal cancer [21]. To analyze whether the anti-tumor therapy induces changes in the levels of suppressor cells, we compared ex vivo in peripheral blood in a group of patients with BC (*n* = 12), the levels of Tregs, MDSC, and DCs before and after three cycles of antitumor therapy (Fig. 1b). Once baseline levels of each population in peripheral blood from healthy donors were established (HD, *n* = 10), these were compared with those from patients with BC before and after treatment (*n* = 12). As shown in Fig. 1c, the ex vivo analysis of Tregs, MDSCs and DC (both myeloid and plasmacytoid), present in PBMCs of patients before and after chemotherapy showed no significant differences with the levels detected in HD. Altogether these results suggest that in the cohort of patients analyzed, the levels of different populations of suppressor cells in peripheral blood do not undergo significant changes after chemotherapy.

### Patients with BC exhibit a functional deficiency in dendritic cells that is recovered after treatment

Through different mechanisms, the tumor microenvironment modulates the functional capacity of T and APC compartments [8, 20]. This tumor microenvironment in BC affects the maturation capacity of DCs [20, 22] and function of T cells [23]. However, it is unknown if chemotherapy with A/C restores the responsiveness of T and APC compartments and whether this can be assessed in peripheral blood of treated patients. To evaluate the effect of anti-tumor therapy on the functional capacity of the APC compartment, we established an in vitro system in order to analyze the expression of several maturation markers on DC derived in situ from monocytes [11] and on plasmacytoid and myeloid DCs present in PBMCs (cells CD123+ or CD11c + within Lin-1-/HLA-DR+ population respectively – Fig. 2a), after stimulation with a cocktail of pro-inflammatory cytokines [12]. As expected, after stimulation of PBMCs with cytokines, monocyte-derived DCs of HD showed a positive response to the pro-inflammatory stimulus that was evidenced by increased expression of CD83, CD86, and CCR7 in comparison with immature DCs (Fig. 2b). We choose the expression of CD83 as a key marker to identify mature DCs; then we compared the delta percentage of mature DC minus the percentage of immature DCs in HD and BC patients before and after treatment (Fig. 2c). In contrast to what was observed in DC of HD, in the patient group, monocyte-derived DCs and myeloid DC before therapy exhibited a reduced expression of CD83 in response to the maturation stimuli (*p* < 0.001 and *p* < 0.01, respectively) that was restored in myeloid DC after chemotherapy (Fig. 2c). Interestingly, it was found that in response to a cytokine cocktail, the expression of CD83 in plasmacytoid DCs of patients (either before or after chemotherapy) and HD was rather similar (Fig. 2c). Besides the measurement of CD83 to evaluate the functionality of the DC we also measured the secretion of IL-12p70 (IL-12) in culture supernatants of PBMCs stimulated with the combination of pro-inflammatory cytokines described by Mailliard et al. [12]. While IL-12 was clearly detected in cells of HD in the presence of the cytokine cocktail, a significant reduction in the secretion of IL-12 in cells of patients before chemotherapy was observed, and furthermore, the production of IL-12 was significantly recovered after three doses of A/C (Fig. 2d). Altogether, these results show a remarkable defect in the APC compartment of patients before therapy that is recovered after chemotherapy.

### In BC patients, T cells have impairment in TCR internalization and expression of activation markers

As observed in DCs, we hypothesized that T cells could also have a functional defect in BC patients. To evaluate the capacity of T cells to respond in vitro, we stimulated patients’ and HD’s PBMCs with anti-CD3/CD28/CD2 beads for 24 h. As expected, after the in vitro stimulation, T cells from HD showed efficient TCR internalization, evidenced by the reduction of CD3 MFI (Fig. 3a). Paired analyses demonstrated that the internalization elicited by the stimulus was statistically significant in the healthy individuals examined (Fig. 3b left panel). Fig. 3b right panel, shows that TCR internalization in BC patients before therapy was compromised (*p* < 0.05). Following TCR internalization, several activation signals on T cells like increased expression of the alpha chain of the IL-2 receptor (CD25), CD154 (CD40L) and CD69 are associated with this phenotype [24, 25]. We evaluated the activation phenotype of CD3+ T lymphocytes in response to the in vitro stimulation in HD; we found an increased expression of CD25 (not shown), CD154, and CD69 in response to anti-CD3/CD28/CD2 beads (Fig. 3c). However, when we compared the delta of MFI (MFI of stimulated cells minus MFI of unstimulated cells) in BC patients before and after chemotherapy, we observed a small difference in the expression of CD25 (*p* = 0.081) between HD and BC patients before treatment (Fig. 3d, left panel). The expression of CD69 show a significant impairment in BC before and after chemotherapy compared to the expression levels of HD (Fig. 3d, central panel). The limited expression of CD154 elicited by the stimulus observed in BC patients before therapy compared to HD showed a partial recovery after chemotherapy (Fig. 3d, right panel). Together, these results suggest a dysfunctional capacity of T cells in BC patients before treatment.

### Uncovering the effect of neoadjuvant chemotherapy by multivariable analysis of the immune response in BC patients

The efficient activation of T cells against tumors is a multi-step process that relies not only on the capacity of APC to stimulate T cells via TCR/MHC interactions [26] but also in the ability of activated T cells to stimulate on APCs the expression of costimulatory molecules such CD83 and the production of IL-12 via the stimulation by CD154 (expressed by T cells upon activation) of CD40 on APCs [27, 28]. For these reasons, we propose that the dysfunction of T and APC compartments evidenced here in BC patients are correlated and, furthermore, that by assessing the functional performance of these two compartments is possible to discriminate between BC patients and HD status. Our results so far suggest an associated defect in the functional capacity of APCs and T cells in patients with BC before the anti-tumor therapy in agreement with a diverse array of suppressive mechanisms of tumor cells that hampers immune surveillance of tumors [29]. To clarify different possible correlations that may exist between the cells responsible for the immune response against tumors, using the proposed in vitro model, we compared several immunological readouts in HD and cancer patients. To do this, we used multivariate analysis (factor analysis with Principal Component Analysis - PCA), to simultaneously evaluate different parameters assesed in PBMCs. We selected the variables (Additional file 1: Table S1) that best describe the behavior of the samples by the matrix of components of each variable in the PCA (Additional file 2: Figure S1A). Examining the scores of the PCA in the samples, we observed a clear separation between HD and BC patients before anti-tumor treatment; samples of some patients after chemotherapy have an intermediate behavior between HD and the same patient before receiving treatment (Additional file 2: Figure S1B). This result would suggest that this in vitro model may be useful to monitor the immune and clinical responses in BC patients along adjuvant chemotherapy. Finally, we compared the sensitivity and specificity of several immunological determinants analyzed to differentiate between HD and BC patients; for this, using ROC curves we examined the area under the curve (AUC) of the internalization of the TCR (Additional file 2: Figure S1C, AUC = 0.67; *p* = 0.05). The multiparametric analysis done by PCA represent the complexity of the immune system involved in the response of BC patients to A/C chemotherapy. The analysis of this complexity with our model suggest that by assessing the functionality of T/APC compartments in blood it is possible to differentiate between HD and BC patients.

### Usefulness of immunological readouts to predict clinical response of tumors to A/C chemotherapy

Predicting clinical response of tumors to neoadjuvant chemotherapy remains a formidable challenge. Despite that molecular testing of TOP2A and the in situ analysis of tumor landscape after neoadjuvant chemotherapy are promising readouts useful to predict tumor response and survival in treated BC patients [3, 30], the usefulness of functional analyses of peripheral blood leukocytes to predict tumor responsiveness to chemotherapy remains unknown. To address this possibility, the immunological readouts of all functional studies performed with peripheral blood leukocytes from patients before chemotherapy were categorized first and then analyzed by a multifactorial categorical analysis (by general estimating equations - GEE). This was done in order to calculate coefficients for each explanatory variable that best fit a model that explain the behavior of a given clinical parameter based on immune readouts (Additional file 3: Table S2). Taking into account three immunological readouts after chemotherapy: CD3 internalization and CD69 expression in T cells and the IL-12 production in DCs, became evident that the explanatory values of CD69 and IL12 (Additional file 3: Table S2) are useful for predicting tumor response to chemotherapy. All three values are associated with the expression of estrogen receptor but not to the expression of progesterone receptors, Her2/neu or KI-67 by the tumor (Additional file 3: Table S2). Altogether, these results suggest that responsiveness of the T and APC compartments and tumor clinical response are two components prompted by chemotherapy that somehow are related.

**Fig. 4:**
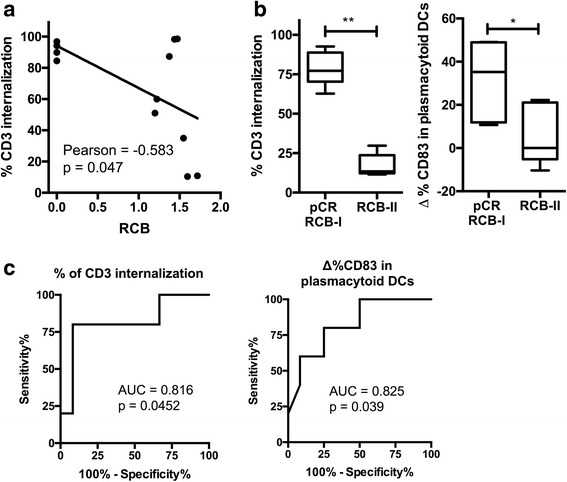
The predictive capacity of immune readouts for clinical response to chemotherapy. **a** Scatter plot of percentage of CD3 internalization vs. residual cancer burden (RCB) index in BC patients after chemotherapy (Pearson correlation = −0.583, *p* < 0.05). **b** Predictive value of TCR (CD3) internalization (left panel) and the delta percentage of CD83 expression in plasmacytoid DCs evaluated before therapy and compared in patients with or without better clinical response (pCR/RCB-I vs RCB-II respectively). **c** ROC curves of TCR internalization (AUC = 0.816, *p* = 0.0452), and delta percentage of CD83 expression in plasmacytoid DCs to predict tumor response (AUC = 0.825, *p* = 0.039). Box and whiskers 10–90%, Pearson correlation test, Mann-Whitney test, * *p* < 0.05, ** p < 0.01

To further confirm this, we examined which immune characteristics are associated with the best fitting of tumor clinical response to chemotherapy. We found that after three doses of chemotherapy with A/C, TCR internalization is correlated with tumor response to the treatment quantified by RCB index (Fig. 4a).

Finally, we assessed the predictive value of immunological readouts (before chemotherapy) to predict beforehand the clinical outcome before treatment, to do this, the performance of readouts before treatment was cross-checked with RCB classification after chemotherapy. Higher levels of TCR internalization (*p* < 0.01), and matured plasmacytoid DCs (*p* < 0.05) were found in patients with better tumor response (pCR or RCB-I) compared with patients with higher RCB (RCB-II) (Fig. 4c). Based on these results, we established the sensibility and specificity of this immunological readout in predicting tumor response to A/C treatment by doing a ROC curve. We found that the ROC curves of CD3 internalization and DC maturation have a high AUC (0.816 and 0.825 respectively) that discriminate patients who respond from those who do not respond (Fig. 4d). These results let us argue that high levels of mature plasmacytoid DCs and the internalization of CD3 detected in peripheral blood before chemotherapy after in vitro stimuli as useful biomarkers for predicting clinical responsiveness of tumors to primary chemotherapy with A/C in BC patients.

## Discussion

Based on parameters used by us to measure tumor-specific T cells generated in response to anti-tumor therapy [9], in the present study, we monitored a series of immunologic parameters in BC patients’ PBMCs obtained before and after chemotherapy with A/C trying to establish, first, the capacity of neoadjuvant chemotherapy to reestablish immune responsiveness and second, the usefulness of immunological readouts to predict clinical tumor response prior to treatment. We evaluated the expansion and phenotype of Tregs, MDSCs, and DCs present in patients’ PBMCs. These three populations play a critical role in tumor escape of immune surveillance [4, 31]. Statistically significant differences between the levels of Tregs and MDSCs found in samples of patients’ PBMCs (obtained either before or after chemotherapy) with those observed in control’s PBMCs were not found. The fact that these measurements have usually been made in BC patients with advanced disease and not in patients with newly diagnosed primary tumors and before neoadjuvant chemotherapy, as in our case, may explain these results.

MDSCs are a heterogeneous population of myeloid cells that accumulate in cancer patients inhibiting T cell-mediated immune responses through the production of NO, Arginase and reactive oxygen and nitrogen species, which foster tumor infiltration by Tregs [32, 33]. By studying the role of MDSCs in inhibiting immune surveillance of BC tumors, Verma et al., reported the increase of MDSCs from two different sources: monocyte-derived (cells CD11b + CD14+ CD124+ CD33+) and PMN-derived (cells CD11b + CD14- HLA-DR- CD66b + CD124+ CD15+) in peripheral blood of BC patients in neoadjuvant chemotherapy [34]*.* On the other hand, Yu et al. [35], reported an increase of cells Lin- HLA-DR- CD14- CD15- CD13+ CD33+ that produce IDO in BC patients with advanced tumors. Using the same markers employed by Yu et al. (except for IDO), we did not find differences in the amounts of MDSC Arginase + in BC patients before chemotherapy in comparison with controls. The analysis of a complex population such as MDSC in different studies using different sets of markers makes difficult the comparison between studies.

On the other hand, the impact of A/C chemotherapy in the levels of MDSCs and Tregs has been a matter of debate. The use of A/C was associated with a significant increase of MDSCs in the blood of newly diagnosed BC cancer patients correlated with disease stage and metastatic tumor burden [36]. In contrast, a more recent study shows a decrease in the levels of MDSCs and Tregs in blood attributable to the cytotoxic effect of A/C on these cells [34]. After three cycles of A/C, we did not observe variations in levels of MDSCs or Tregs (neither CD127- nor FoxP3+). The difference between our results and those of others may be explained because the measurement of MDSCs and Tregs in blood pre- and post- chemotherapy have not been previously analyzed. In short, the contrasting results regarding the behavior of MDSCs and Tregs during anti-tumor therapy argues for the need for standardized methods for monitoring these two cell populations in patients during treatment.

IL-12 produced by DCs is a key point in cancer immunotherapy as it promotes CTLs that secrete IFN-γ a cytokine with recognized anti-tumor activity [37]. This evidence suggests that evaluating the immune competence of DCs to produce IL-12 and to mature in response to a pro-inflammatory stimulus is useful to assess the immune surveillance of tumors. Very recently a whole-blood assay that was used for monitoring the immune competence in cohorts of healthy women and BC patients at different progression stages prior any treatment evidenced unresponsiveness of patients’ BDCA3 DCs to interferon alpha [38]. In another study, Della Bella et al., reported a decrease in the absolute number of myeloid DCs in whole blood of BC patients’ ex vivo [20]. This reduction that was associated with a decrease in CD119 (IFN-γR) and increased expression of CD83 without altering the expression of CD80 and CD86 in response to LPS was correlated with the severity of BC. Although we did not observe marked differences in percentages of DC populations among HD, and BC patients pre- and post-treatment, after three doses of chemotherapy we found a substantial recovery of CD83 expression and production of IL-12 in response to a cocktail of cytokines used by Mailliard et al., [12] to derive type I alpha DCs in situ [11]. An increased production of IL-12 was detected after tumor removal in the study by Della Bella et al. [20], this and that the clinical tumor response to A/C correlates with the production of IL-12 and CD83 expression by DCs in the present study suggest that the responsiveness of DCs to the pro-inflammatory stimuli used here is useful for monitoring the recovery of immune surveillance by DCs during neoadjuvant treatment with A/C. In the same vein, results of preclinical studies in mice show that the A/C promotes recovery of immune surveillance associated with antigen presentation, increased expression of CD83 and IL-12 production by DCs [39]. However, it is possible that IL-12 production by DCs has different prognostic value depending on the state of the disease, our results suggest that in early stages of treatment it promotes the recovery of the immune-surveillance and a favorable clinical response compared to its production after treatment that apparently favors tumor relapse [38].

We observed a more efficient TCR internalization and the CD154 (CD40L) expression on T cells after chemotherapy. CD154 is expressed on both CD4 and CD8 T cells upon TCR stimulation. However, the consequence of activated CD4 Th1 cells expressing CD154 is better known [40, 41]. In this regard, we speculate that the recovery of CD154 by Th1 cells may foster CD8 surveillance in BC patients treated with AC by promoting competent DCs after cognate CD40/CD40L interaction that probably stimulates IL-12 secretion as well as the up-regulation of adhesion and co-stimulatory molecules by DCs (e.g., CD83), all of which have been shown to occur after CD40 cross-linking on these two cell types [40–48]. On this perspective, the responsiveness of the T and APC compartments after therapy observed in our patients argues in favor that neoadjuvant therapy reestablishes the cross talk between these compartments and that this is essential for immune surveillance (Additional file 4: Figure S2).

By multivariate PCA analysis, it was possible to integrate TCR internalization, CD83 expression and IL-12 production by mature DCs, with some immunological readouts (Additional file 1: Table S1). Despite neither parameter when were considered individually allows to discriminate between HD and patients, the PCA allowed us to segregate HD individuals from donors in the patient group clearly. In this regard, it is evident that after treatment, the behavior of variables in some patients becomes like those observed in the control group (HD). Finally, by using ROC curves, the TCR internalization allowed us to differentiate the immune response between HD and patients. Taken together these results lead to propose that the recovery of crosstalk between T and APC compartments induced by A/C therapy reflects the restoration of immune surveillance and is a good prognostic factor in BC patients treated with neoadjuvant A/C (Additional file 4: Figure S2).

Finally, it is of great interest to define biomarkers able to predict clinical response to chemotherapy in BC patients, in this regard candidate biomarkers are tumor infiltration by CD8+ T cells [3] and TFH [49] and in situ expression of markers such as HMGB-1 and autophagy [50]. We propose that the proper TCR internalization and IL-12 production in response to treatment are potential biomarkers to predict tumor size reduction after three months of chemotherapy. The correlation between clinical response and ex vivo levels prior therapy of plasmacytoid DC CD83+ (a cell that produces type-I IFN important to activate anti-tumor responses) suggests this marker as useful for predicting clinical response to treatment. This result is consistent with the description of a type I IFN-related signature that predicts clinical responses to anthracycline-based chemotherapy in several independent cohorts of BC patients [51].

## Conclusion

In summary, our results argue for the usefulness of in vitro assays using whole blood [38] or PBMCs from BC patients to monitor the responsiveness of T and APC compartments during treatment and to identify predictive markers of favorable clinical tumor response.

## Additional files

Additional file 1: Table S1. Variables selected for PCA. (DOCX 39 kb)

Additional file 2: Figure S1. Unresponsiveness of T cell and APC compartments are correlated in BC patients*.* (A) Principal component analysis (PCA) of several immunological readouts (Additional file 1: Table S1) selected after Varimax rotation, and two principal components (PC) were extracted and show the variable loadings of rotated component matrix, and (B) dot plot of PC score of HD (half-filled circles), and patients before (white box) and after (black box) neoadjuvant chemotherapy. The dashed line represents an axis that separates BC patients from HD. KMO and Bartlett’s Test 0.681 *p* = 0.005. (C) Receiver operating characteristic (ROC) curves, to differentiate HD vs. BC patients before therapy using TCR internalization by MFI CD3 (left curve, AUC 0.67 *p* = 0.05). (TIFF 1302 kb)

Additional file 3: Table S2. Association between immunological readouts in peripheral blood and clinicopathologic factors of BC patients. (DOCX 81 kb)

Additional file 4: Figure S2. Immunomonitoring model of breast cancer patients treated with chemotherapy with A/C. (A) In patients with established BC, the immune system could not control the tumor growth phase called immune escape. Tumor cells exhibit a decreased amount of MHC class I and release suppressive cytokines such as IL-10 and TGF-β, there is a greater frequency of suppressor cells like MDSCs (that secrete arginase), Tregs, and plasmacytoid DCs or immature DCs (with high levels of IDO). These suppressor cells favor a weak cytotoxic T cells activation and inhibition of function of T helper CD4+ cells by suppressive cytokines such as IL-10. (B) In BC patients who are treated with chemotherapy A/C, the proposed immunomonitoring system can evaluate the restoration of immunosurveillance of tumors by promoting the immune response by inducing ICD in tumor cells with the release of DAMPs (CRT, HMGB1, and ATP) and apoptotic bodies that are recognized by immature DCs. This recognition induces maturation of DCs with increased expression of CD80, CD83, CD86, and antigen cross-presentation favoring the recognition of these antigens by T cells. Stimulated T cells induce the production of IL-12 by the interaction CD154 with the CD40 receptor on APCs and thus assisting in the production of IFN-γ providing helper activity to CTLs to attack the remaining tumor cells. (TIFF 6599 kb)
