# Supplementary material for: Monitoring the responsiveness of T and antigen presenting cell compartments in breast cancer patients is useful to predict clinical tumor response to neoadjuvant chemotherapy
Source: BMC Cancer. 2018 Jan 15;18:77. doi: 10.1186/s12885-017-3982-1 (PMC5769526; doi:10.1186/s12885-017-3982-1)
Supplement: Supplementary file 1 — Variables selected for PCA. (DOCX 39 kb) [file 12885_2017_3982_MOESM1_ESM.docx]

**Table S1. Variables selected for PCA**

| % CD25- CD69- |
| --- |
| % CD25- CD69+ |
| % CD154- CD69+ |
| MFI CD69 |
| MFI CD154 |
| % CD3 internalization |
| % Myeloid CD83 |
| % DCs CD83 |
| % CD154+ CD69+ |
| Tumor size (cm^2^) |
| % CD25+ CD69+ |
| MFI CD25 |
| MFI CD3 |
| IL12p70 |
| % Myeloid DCs |
