# Supplementary figures and images for: Monitoring the responsiveness of T and antigen presenting cell compartments in breast cancer patients is useful to predict clinical tumor response to neoadjuvant chemotherapy
Source: BMC Cancer. 2018 Jan 15;18:77. doi: 10.1186/s12885-017-3982-1 (PMC5769526; doi:10.1186/s12885-017-3982-1)

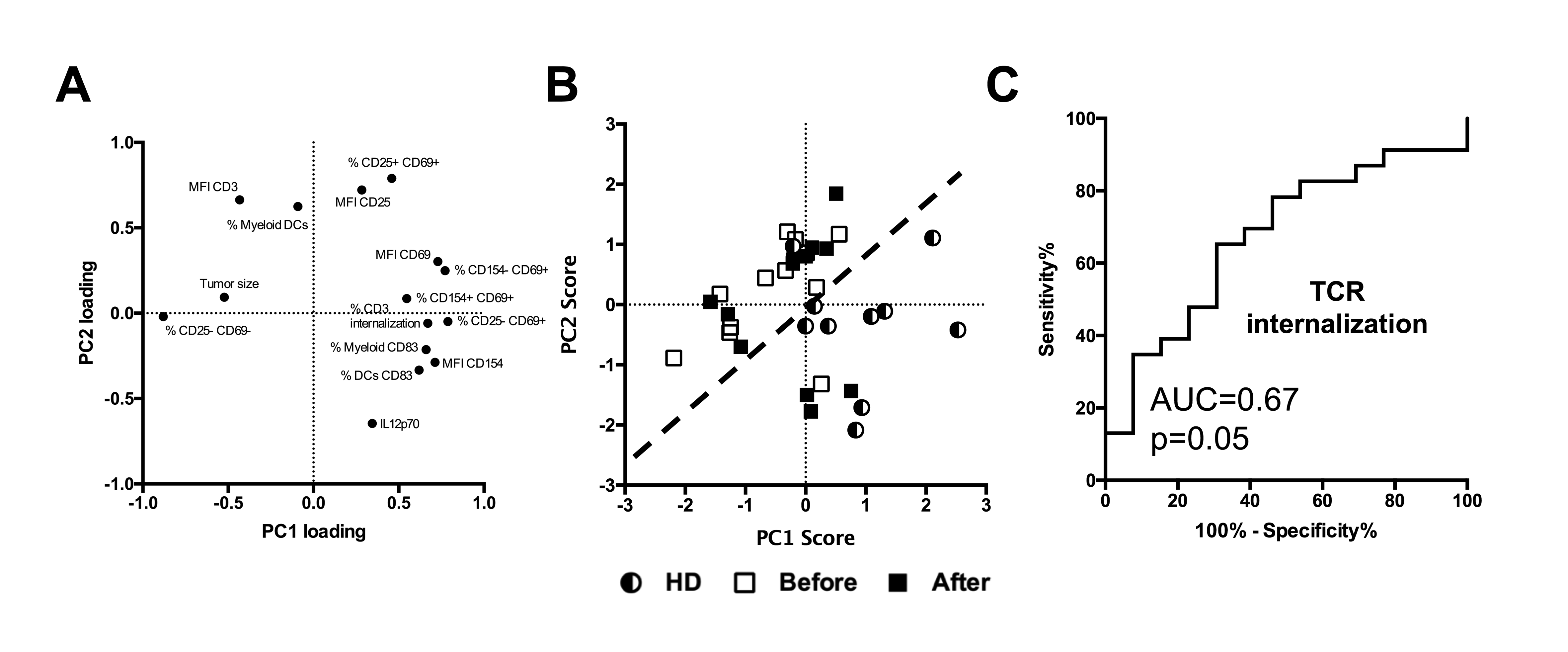

Supplement: Supplementary file 2 — Unresponsiveness of T cell and APC compartments are correlated in BC patients. (A) Principal component analysis (PCA) of several immunological readouts (Additional file 1: Table S1) selected after Varimax rotation, and two principal components (PC) were extracted and show the variable loadings of rotated component matrix, and (B) dot plot of PC score of HD (half-filled circles), and patients before (white box) and after (black box) neoadjuvant chemotherapy. The dashed line represents an axis that separates BC patients from HD. KMO and Bartlett’s Test 0.681 p = 0.005. (C) Receiver operating characteristic (ROC) curves, to differentiate HD vs. BC patients before therapy using TCR internalization by MFI CD3 (left curve, AUC 0.67 p = 0.05). (TIFF 1302 kb) [file 12885_2017_3982_MOESM2_ESM.tif]

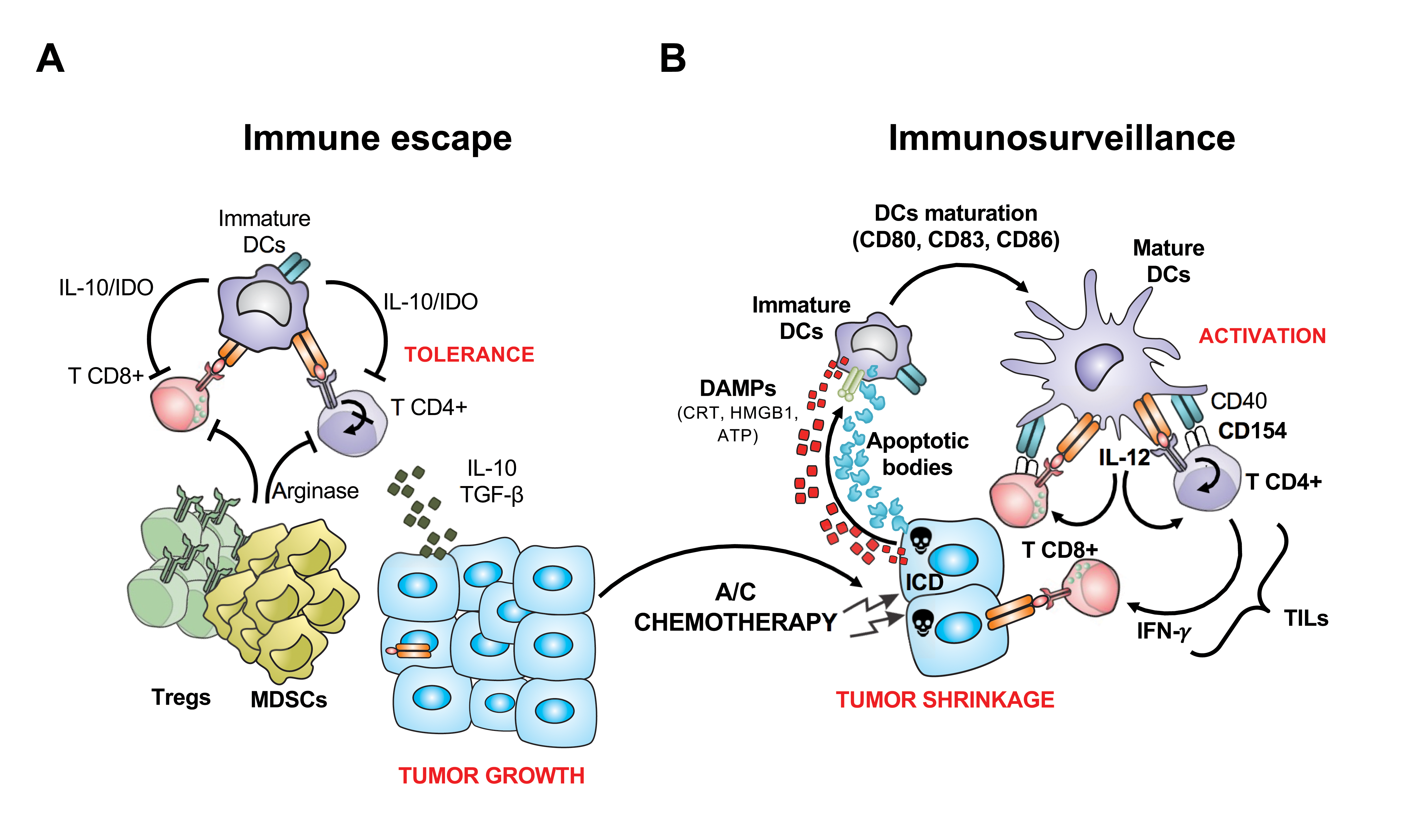

Supplement: Supplementary file 4 — Immunomonitoring model of breast cancer patients treated with chemotherapy with A/C. (A) In patients with established BC, the immune system could not control the tumor growth phase called immune escape. Tumor cells exhibit a decreased amount of MHC class I and release suppressive cytokines such as IL-10 and TGF-β, there is a greater frequency of suppressor cells like MDSCs (that secrete arginase), Tregs, and plasmacytoid DCs or immature DCs (with high levels of IDO). These suppressor cells favor a weak cytotoxic T cells activation and inhibition of function of T helper CD4+ cells by suppressive cytokines such as IL-10. (B) In BC patients who are treated with chemotherapy A/C, the proposed immunomonitoring system can evaluate the restoration of immunosurveillance of tumors by promoting the immune response by inducing ICD in tumor cells with the release of DAMPs (CRT, HMGB1, and ATP) and apoptotic bodies that are recognized by immature DCs. This recognition induces maturation of DCs with increased expression of CD80, CD83, CD86, and antigen cross-presentation favoring the recognition of these antigens by T cells. Stimulated T cells induce the production of IL-12 by the interaction CD154 with the CD40 receptor on APCs and thus assisting in the production of IFN-γ providing helper activity to CTLs to attack the remaining tumor cells. (TIFF 6599 kb) [file 12885_2017_3982_MOESM4_ESM.tif]
