# Supplementary material for: Monitoring the responsiveness of T and antigen presenting cell compartments in breast cancer patients is useful to predict clinical tumor response to neoadjuvant chemotherapy
Source: BMC Cancer. 2018 Jan 15;18:77. doi: 10.1186/s12885-017-3982-1 (PMC5769526; doi:10.1186/s12885-017-3982-1)
Supplement: Supplementary file 3 — Association between immunological readouts in peripheral blood and clinicopathologic factors of BC patients. (DOCX 81 kb) [file 12885_2017_3982_MOESM3_ESM.docx]

**Table S2. Association between immunological readouts in peripheral blood and clinicopathologic factors of BC patients.**

| **Clinical characteristic** | **Wald (Chi)^2^** | **Model variables**  **(Immunological readout)** | ***Coefficient*** | ***p value*** | ***95% CI*** |
| --- | --- | --- | --- | --- | --- |
| KI-67 | 1.49 | % of CD3 internalization | -.0293 | 0.332 | -.0886 to .0299 |
|  |  | MFI of CD69 | .0005 | 0.621 | -.0015 to .0025 |
|  |  | IL-12p70 concentration (pg/mL) | -.0387 | 0.281 | -.1093 to .0317 |
| HER2/neu | 1.72 | % of CD3 internalization | -.0407 | 0.194 | -.1022 to .0207 |
|  |  | MFI of CD69 | .0010 | 0.382 | -.0012 to .0031 |
|  |  | IL-12p70 concentration (pg/mL) | -.0090 | 0.571 | -.0401 to .0221 |
| Tumor response | 6.1 | % of CD3 internalization | .0067 | 0.780 | -.0403 to .0536 |
|  |  | MFI of CD69 | -.0029 | **0.061^*^** | -.0058 to .0001 |
|  |  | IL-12p70 concentration (pg/mL) | -.0110 | **0.094^*^** | -.0239 to .0019 |
| Estrogen receptor | 9.68 | % of CD3 internalization | -.0867 | **0.002^*^** | -.1428 to -.0305 |
|  |  | MFI of CD69 | .0039 | **0.004^*^** | .0012 to .0066 |
|  |  | IL-12p70 concentration (pg/mL) | .0120 | **0.002^*^** | .0042 to .0198 |
| Progesterone receptor | 1.23 | % of CD3 internalization | -.0261 | 0.323 | -.0779 to .0256 |
|  |  | MFI of CD69 | .0003 | 0.732 | -.0016 to .0023 |
|  |  | IL-12p70 concentration (pg/mL) | -.0038 | 0.663 | -.0208 to .0132 |

Multiple regression analysis – GEE, * p<0.1 was considered significant
